# 

**Published:** 1871-06

**Authors:** 


Vol. XII. JUNE, 1871. No. 6.

### CHICAGO Medical Examiner,

N. S. DAVIS, M.D., Editor,

AND

F. H. Davis, M.D., Assist.-Editor.

TERMS, $3.00 PER ANNUM, IN ADVANCE.

CHICAGO:

ROBERT FERGUS' SONS, PRINTERS,

12 CLARK AND 242—248 ILLINOIS STREET.

1871.

